# The impact of cataract surgery on tear film physiology: signs and symptoms, progression and treatment

**DOI:** 10.3389/fmed.2025.1559323

**Published:** 2025-08-19

**Authors:** Alessia Nuzzi, Davide Tibaldi, Raffaele Nuzzi

**Affiliations:** Eye Clinic, San Luigi Gonzaga University Hospital, University of Turin, Orbassano, Italy

**Keywords:** tear film, dry eye, dry eye disease, cataract, cataract surgery

## Abstract

**Purpose:**

This study aimed to revise data published in the literature on the effects of cataract surgery on tear film characteristics, in relation to personal clinical surgical experience.

**Methods:**

A search was undertaken using PubMed (all years). Search terms included ‘cataract surgery’, ‘phacoemulsification’, ‘cataract extraction’, and ‘manual small-incision cataract surgery’, combined at first with ‘ocular surface’ and ‘lacrimal film’. Second, we combined terms as ‘cataract surgery’, ‘phacoemulsification’, ‘cataract extraction’, ‘manual small-incision cataract surgery’ with ‘dry eye disease (DED)’ and ‘dry eye syndrome’. Third, we combined terms such as ‘cataract surgery’, ‘phacoemulsification’, ‘cataract extraction’, and ‘manual small-incision cataract surgery’ with ‘conjunctival sensitivity’ and ‘sensitivity of the conjunctiva’. Finally, we combined terms as ‘cataract surgery’, ‘phacoemulsification’, ‘cataract extraction’, and ‘manual small-incision cataract surgery’ with ‘epiphora’ and ‘excessive tear’. Relevant in-article references not returned in our searches were also considered.

**Results:**

We analyzed collected data regarding DED characteristics and management of this condition related to cataract surgery. The relationship between ocular surface signs and symptoms and cataract surgery appears to be strong; indeed, TBUT, Schirmer’s test, and OSDI scores are abnormal as early as 2 weeks after cataract surgery; however, there is conflicting evidence concerning the duration of these alterations and the restoration of the preoperative conditions, generally within 6 months after surgery. An increased risk of DED after cataract surgery is associated with pre-existing MGD. The chosen surgical procedure and pre- and postoperative pharmacological management are also key points in determining the extent of postoperative DED. Finally, no remarkable evidence was found regarding the association of “cataract surgery” with “hyperlacrimation” or “conjunctival sensitivity.”

**Conclusion:**

The available evidence is discrepant regarding the onset, progression, and management of this condition. However, the association between cataract surgery and the occurrence of DED thereafter is well documented. Multicenter randomized trials are needed to strengthen the already published data, to investigate these divergencies, and to establish diagnostic-therapeutic protocols to manage this condition.

## 1 Introduction

**Figure 1:**
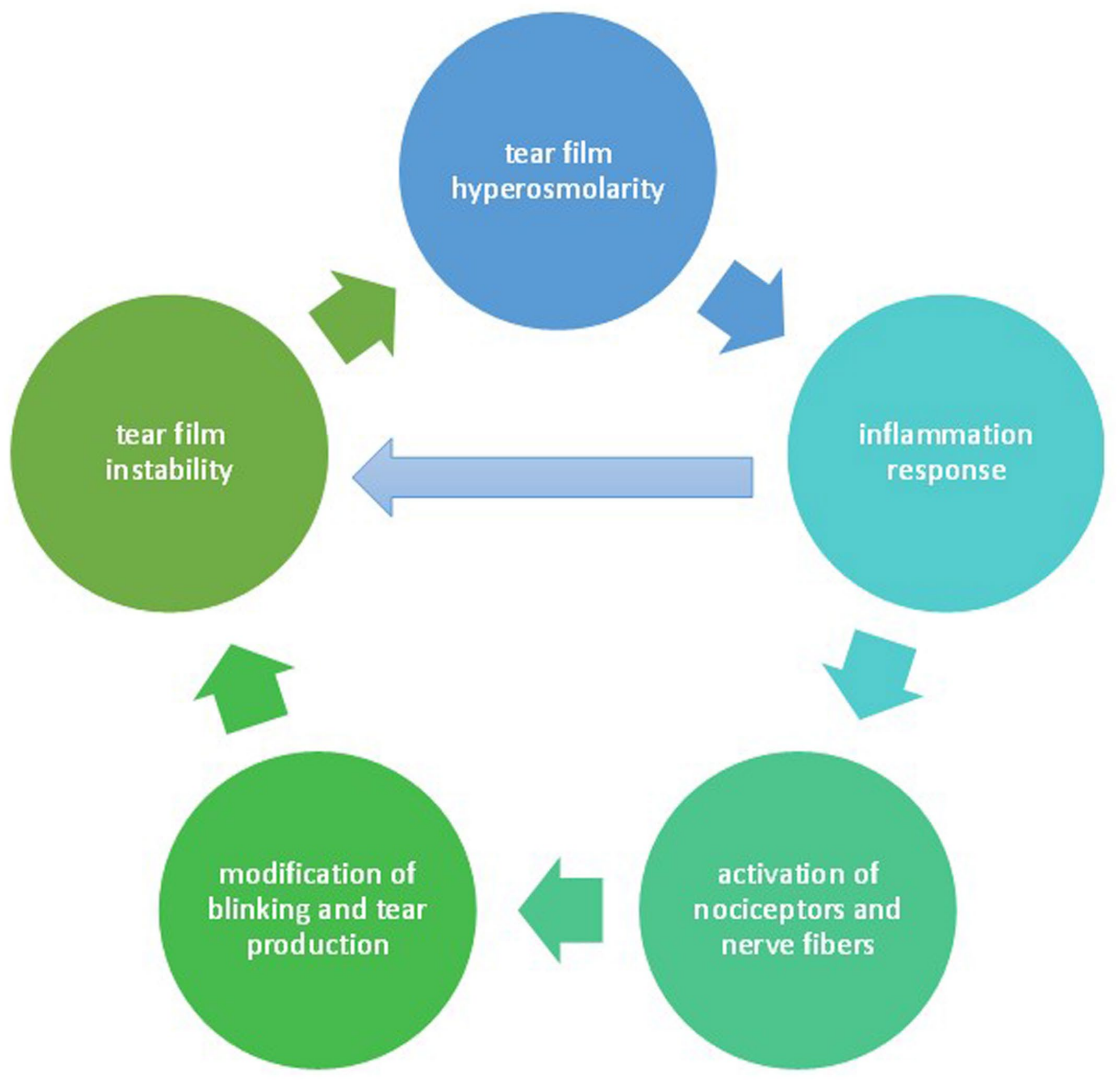
Dry eye disease pathology.

An estimated 95 million people worldwide are affected by cataract, and it is the leading cause of blindness in middle-income and low-income countries nowadays (1). However, it is also responsible for visual impairment in developed countries: in the United States as a whole, an average of 7.5 million patients with cataract are estimated to require surgery. This procedure is one of the most frequently performed surgical operations, and given the progressive aging of the world’s population, the number of interventions is set to rise (2). Another disorder that is growing in parallel is dry eye disease (DED): according to the Tear Film and Ocular Surface Society (TFOS) Dry Eye Workshop II (DEWS II), the definition of DED is “a multifactorial disease of the ocular surface characterized by a loss of homeostasis of the tear film, and accompanied by ocular symptoms, in which tear film instability and hyperosmolarity, ocular surface inflammation and damage, and neurosensory abnormalities play etiologic roles” (3). Therefore, it consists of the multifactorial dysfunction of the functional lacrimation unit. This complex consists of the lacrimal gland, the ocular surface (which in turn consists of the cornea, tear film, and meibomian glands), the eyelids, as well as sensory and motor innervation. Any disruption in the integrity of any of these structures can result in dry eye syndrome (DES), which is characterized by ocular discomfort or burning, foreign body sensation, blurred vision, tearing, and light hypersensitivity. At the eye examination, signs such as conjunctival hyperemia, eyelid margin irregularity, and tear film changes associated with positive Schirmer’s test, reduced Tear Break Up Time (TBUT), and/or meibomian gland dysfunction (MGD) can be identified. Moreover, such dryness can result in superficial punctate keratopathy, which can be detected by the fluorescein staining test. Signs and symptoms are summarized in Table 1. However, the discrepancy between patient-reported symptoms and detectable signs is peculiar to this condition (4, 5). The causes of DED are various, but dealing with the characteristics and mechanisms is beyond the scope of this discussion. They are schematically shown in Figure 1. The pivotal treatment consists of artificial tears instillation and eyelid hygiene. Other options available are topical anti-inflammatory eye drops, mainly corticosteroids, non-steroidal anti-inflammatory drugs (NSAIDs), and cyclosporine, or in more severe cases, there are parasurgical (tear duct occlusion by plugs, therapeutic scleral contact lens placement) and surgical (amniotic membrane application, tarsorrhaphy) solutions (Table 2). What has been observed is the occurrence or worsening of DED in patients in the period following cataract surgery, and this disease profoundly affects the quality of vision as well as the quality of life (QoL) (6, 7). Indeed, studies showed that patient satisfaction with cataract surgery is closely associated with DED symptoms, rather than objective measures of postoperative visual acuity and signs of DED (8). The aim of our study was to collect data from the literature on changes and/or alterations of the tear film in patients undergoing cataract surgery and how to manage this condition in the pre- and post-operative period.

**Table 1:** Signs and symptoms of DED.

Symptoms	Signs
Ocular discomfort	Conjunctival hyperemia
Burning	Eyelid margin irregularity
Foreign body sensation	Positive Schirmer’s test
Blurred vision	Reduced Tear Break Up Time (TBUT)
Tearing	Meibomian gland dysfunction (MGD)
Light hypersensitivity	Fluorescein corneal staining

**Table 2:** DED management.

DED management	
First line therapy	Tear substitutes and eyelid hygiene
Second line therapy	Topical medications, e.g., corticosteroids, non-steroidal anti-inflammatory drugs (NSAIDs) and cyclosporine A (CsA)
Third line therapy	Tear duct occlusion by plugs, therapeutic scleral contact lens
Fourth line therapy	Amniotic membrane placement, tarsorrhaphy

## 2 Methods

A search was undertaken using PubMed (all years) and Google Scholar (all years). Published articles in English were preferentially selected. Relevant in-article references not returned in our research were also considered. We used the terms [“cataract surgery,” “cataract surgeries,” “cataract extraction,” “cataract extractions,” “phacoemulsification,” “phacoemulsifications,” “MSICS,” “manual small-incision cataract surgery,” and “manual small-incision cataract surgeries”] to identify the cataract extraction procedure in our review. We first investigated the relationship between cataract surgery and normal ocular tear film. We used the following search string: [(“Cataract surgery OR cataract surgeries OR cataract extraction OR cataract extractions OR phacoemulsification OR phacoemulsifications OR MSICS OR manual small-incision cataract surgery OR manual small-incision cataract surgeries) AND (Ocular surface OR Ocular surface OR Ocular surfaces OR lacrimal film OR lacrimal film)]. We found 503 results. We excluded those publications that were not relevant. We selected 30 articles to describe the relationship between cataract surgery and normal tear film. Then we studied the relationship between cataract surgery and DED. We used the following search string: [(Cataract surgery OR Cataract surgeries OR Cataract extraction OR Cataract extractions OR Phacoemulsification OR Phacoemulsifications OR MSICS OR manual small-incision cataract surgery OR manual small-incision cataract surgeries) AND (dry eye disease OR dry eye diseases OR dry eye OR dry eyes OR DED OR Dry eye syndromes OR Dry eye syndrome)]. We found 305 results. We excluded those publications that were not relevant. We selected 55 articles to describe the relationship between cataract surgery and dry eye disease. Third, we investigated the relationship between cataract surgery and hyperlacrimation. We used the following search string: [(Cataract surgery OR Cataract surgeries OR Cataract extraction OR Cataract extractions OR Phacoemulsification OR Phacoemulsifications OR MSICS OR manual small-incision cataract surgery OR manual small-incision cataract surgeries) AND (epiphora OR Excessive Tearing OR Over tearing OR Over tearings)]. We found 165 results, but none revealed remarkable results on the association between cataract surgery and hyperlacrimation. Finally, we investigated the relationship between cataract surgery and conjunctival sensitivity. We used the following search string: [(Cataract surgery OR Cataract surgeries OR Cataract extraction OR cataract extractions OR phacoemulsification OR phacoemulsifications OR MSICS OR manual small-incision cataract surgery OR manual small-incision cataract surgeries) AND (conjunctival sensitivity OR conjunctival sensitivities OR sensitivity of the conjunctiva OR sensitivity of the conjunctivas)]. We found 165 results, but none revealed remarkable results on the association between cataract surgery and conjunctival sensitivity, as in the case of hyperlacrimation.

## 3 Results

Studies have indeed shown that the lacrimal functional unit is altered secondary to cataract surgery. However, incidence rates are different depending on the studies conducted: they range from 9 (9) to 100% (10). Recent studies found that 33% of patients develop DED after surgery (11), while another study reported a 37.4% incidence of DED after phacoemulsification (12). Sahu et al. denoted an onset of dry eye symptoms following phacoemulsification surgery, and the preoperative levels were not achieved until 2 months after the procedure (13). Another study found symptoms and signs of DED occurred as early as 7 days post-phacoemulsification, and the severity pattern improved over time (14). Considering this, a preoperative ophthalmological evaluation, focusing especially on the ocular surface, is necessary to optimize psychological and clinical outcomes of cataract surgery (15). Therefore, a correct diagnostic–therapeutic approach is the key to improving the quality of life of all patients, no one excluded (16), in particular to prevent and/or improve discomfort and accelerate post-operative healing (17). The most important parameters for assessing the health of the ocular surface are corneal sensitivity evaluation, tear film osmolarity, tear break-up time (TBUT), ocular surface staining, the state of the meibomian glands, the goblet cell density, and the quantity of tears excreted (Schirmer’s test); each of these factors has been considered and studied in recent years. Each of these parameters will be described in the following paragraphs separately.

### 3.1 Corneal sensitivity

Many studies document loss of corneal sensitivity after cataract surgery related to the incision of corneal nerve fibers. In contrast, data on the time required to restore this sensitivity to preoperative levels and the actual full recovery of this aspect are discordant. Oh et al. showed a corneal sensitivity decrease 1 day postoperatively, which returned to almost preoperative levels within 1 month, although Schirmer’s test results were not very different from preoperative ones (18). Similarly, Kim et al. observed a restoration of corneal nerve fibers to preoperative values 3 months after surgery. On the other hand, Khanal et al. reported corneal sensitivity remains altered at 3 months after surgery, not recovering to pre-operative values (19). In agreement with the above-mentioned results, Lyne demonstrated irreversible loss of corneal sensitivity at the corneal incision 2 years after surgery in 90% (20).

### 3.2 Goblet cell density (GCD)

The state of the mucin layer influences tear film stability by reducing evaporation (21). Mucins are produced by conjunctival goblet cells, and their reduced density (goblet cell density, GCD) is associated with DED occurrence (22). Oh et al. noted a decrease in GCD, which was correlated with operative time, and it had not recovered 3 months after cataract surgery. Therefore, microscopic ocular surface damage during cataract surgery seemed to be one of the pathogenic factors that cause ocular discomfort and dry eye syndrome after cataract surgery (18). This reduction in GCD was also noted by Kholi et al., whose prospective study aimed to analyze the occurrence of signs and symptoms of DED post-cataract surgery (23). Furthermore, it would be influenced by post-operative topical therapy: as a matter of fact, corticosteroids increase GCD (24), while NSAIDs reduce it (25).

### 3.3 Schirmer’s test, tear break up time (TBUT) results, and corneal fluorescein staining

The Schirmer test measures the quantity of tears secreted (normal values above 15 mm in 5 min), while the tear break-up time (TBUT, normal values above 10 s) is an index of tear film stability. Both are reduced in cases of DED, and many studies have shown altered values after cataract surgery. Kholi et al. described abnormal Schirmer’s test results and TBUT values in 48% of patients at 2 weeks after cataract surgery (23). On the other hand, in the following 6 weeks, recovering trends of DED symptoms were seen. They also observed that corneal nerve damage due to corneal incision may reduce tear production and affect physiological evaporation, which can lead to inflammation of the ocular surface (23). This inflammation would lead to the secretion of inflammatory cytokines and chemokines, which in turn would cause cell damage and inflammation, generating a vicious circle (23). Zamora et al. showed how the Ocular Surface Disease Index (OSDI) score increased significantly 1 week postoperatively, while TBUT and Schirmer’s test values decreased 1 month after surgery. So they demonstrated short-term changes in ocular surface signs and symptoms after phacoemulsification in patients at 1 day, 1 week, and 1 month postoperatively (26). Xue et al. found similar evidence: in their study, dry eye symptoms persisted more than 3 months after surgery. In fact, OSDI scores increased post-operatively and then decreased, while BUT and Schirmer’s test results were reduced in the first month after surgery and then returned to within normal limits (27). Similarly, Liu et al. demonstrated how phacoemulsification significantly alters these parameters, together with corneal fluorescein staining, tear film pattern, and the height of the tear meniscus (28). They also observed that a pre-operative TBUT value < 10 s is a predisposing risk factor for the development of post-surgical DED (28). As with TBUT and Schirmer’s test results, corneal fluorescein staining also characterizes dry eye: it consists of staining the corneal surface with fluorescein or lissamine green to highlight epithelial defects. Staining appears to worsen in the postoperative period (26, 29). On the duration of this worsening, data is conflicting: some studies have demonstrated staining that continues for months, while others have observed staining increases up to 7 days postoperatively and then decreases over the next 2 months (14, 23).

### 3.4 Tear film osmolarity

Another parameter analyzed was tear osmolarity, whose normal value is less than or equal to 290–310 mOsm/L. We found only one creditable study on this topic. Igarashi et al. conducted a study that measured pre- and post-operative tear osmolarity values among various parameters: it increased 4-fold over preoperative values, and compared to other dry eye parameters, this variation persisted over time (30).

### 3.5 Meibomian gland disfunction (MGD)

Another important element in the pathogenesis of DED is meibomian gland dysfunction (MGD): it can be caused by cataract surgery, and morphological changes in the glands are involved in the symptomatic manifestations of DED in these patients (31). About this, Kim et al. confirmed this data: they noticed how MGD (meibomian gland dysfunction) parameters showed deterioration after cataract surgery, and it is correlated to the thinning of the tear film lipid layer (32). Song et al. also revealed that obstructive MGD may be aggravated by cataract surgery in the short term, and it recovered 3 months postoperatively (33). In another study, however, Han et al. found that even 3 months after surgery, alterations to the meibomian glands persisted (34).

### 3.6 Surgical procedures

**Figure 2:**
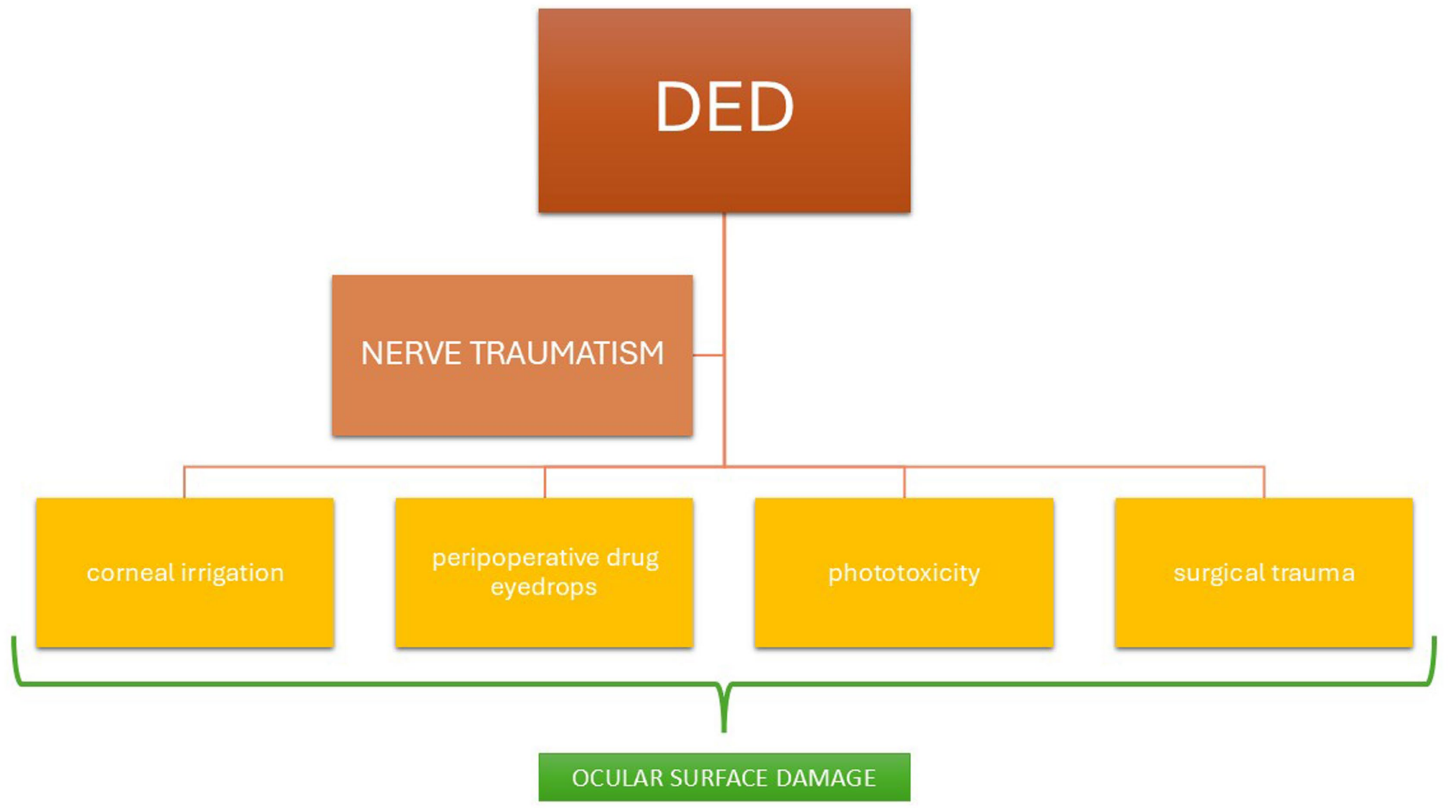
Factors implicated to dry eye disease (DED) onset secondary to cataract surgery.

Surgical technique also plays an important role in the onset and extent of DED following cataract extraction. Therefore, the type of procedure performed is an aspect to be taken into account: Ishrat et al. reported how the incidence of dry eye is higher in small incision cataract surgeries (SICS) than phacoemulsification due to tear film instability (9), while Yu et al. compared phacoemulsification and femto-assisted surgery in terms of the occurrence and/or evolution of DED in the post-operative period. Their study demonstrated that symptoms and signs of DED occurred more frequently or worsened more severely in patients undergoing surgery with the femto-laser than in those operated with phacoemulsification (35), probably due to the traumatic effect induced by femto laser suction on the ocular surface (36, 37). They generally appeared in the immediate post-operative period, disappearing within 3 months post-surgery (36). In contrast, about osmolarity and MM9 values in the tear film, as well as Schirmer’s test results, no statistically significant differences were found between these two surgical procedures (38). However, it is not only the surgical technique that influences the establishment of post-operative DED: He et al. conducted an interesting study on tear film health, observing a beneficial effect of intraoperative viscoelastic application on the corneal surface (39) rather than continuously irrigating with balanced salt solution (BSS); Yusufu et al. also confirmed this in their prospective study (40). Another study was conducted on Mydrane/Fydrane injection during cataract surgery: it seemed to limit the toxicity of the ocular surface and accelerate its restoration compared with topical eyedrops (41). Other perioperative risk factors for DED onset after cataract surgery are operating microscope light exposure time, CDE (cumulative dissipated energy) (13), perioperative anesthetic eyedrops (42), and the povidone-iodine application on the eye surface (43). These factors are schematically shown in Figure 2.

### 3.7 Post-op ocular surface management

There are conflicting results regarding the use of tear substitutes after cataract surgery: Khanal’s study did not find a significant effect of tear substitutes in limiting post-cataract ocular discomfort (19). In contrast, other studies claimed that the use of trehalose/sodium hyaluronate 0.1% (44) or carboxymethylcellulose 0.5% eye drops (45). If MGD is present pre-operatively, the application of lipid-containing artificial tears could be more appropriate (46) associated with preoperative warm compression, eyelid massage (17) or Lipiflow treatment (47): these actions should reduce the incidence of post-operative MGD. Other research supported post-operative instillation of propylene glycol-hydroxypropyl guar (PG-HPG) nanoemulsion lubricant eye drops (48, 49) and 3% Diquafosol (DQS) (50, 51). The use of cyclosporine type A (CsA) also appeared to be effective (52). Compared to previous studies, Kang’s group found a superiority of CsA over carboxymethylcellulose 0.5% (53). The efficacy of preservative-free dexamethasone compared to preserved dexamethasone was also investigated. The former seemed to be associated with fewer incidences of DED (54).

### 3.8 Hyperlacrimation

Our research string revealed no remarkable results on the association between cataract surgery and hyperlacrimation. In these cases, it is recommended to perform an irrigation with antibiotics of the tear ducts (using an antibiotic alone or in combination with corticosteroid) if it occurs only postoperatively. If local peribulbar anesthesia was performed, the clinical status of the eyelids and lacrimal gland should also be evaluated.

### 3.9 Conjunctival sensitivity

Our search string revealed no remarkable results on the association between ‘cataract surgery’ and ‘conjunctival sensitivity’. ‘Conjunctival sensitivity’ must always be correlated to previous toxic ocular therapies (active ingredients, preservatives) and/or to the state of the lacrimal outflow ducts, given that washing of the lacrimal outflow ducts before cataract surgery is no longer routine.

## 4 Discussion

Cataract surgery is constantly evolving and has become more innovative and accessible over the years. On the other hand, patient expectations have increased, both in terms of visual acuity and quality of vision: it is not enough to see well, but the quality must be the best possible. In fact, often the main post-surgical complaint of patients is not so significant visual recovery as ocular discomfort: burning, tearing, and “pins and needles” sensation are challenges every cataract surgeon encounters in the postoperative period of their patients. Therefore, the ophthalmology community is becoming increasingly interested in understanding the mechanisms behind this ocular discomfort, but more importantly, in seeking effective solutions to manage these issues. What has emerged is that the functional lacrimal unit (that is, the apparatus consisting of the lacrimal gland, eyelids, and ocular surface) is altered somewhat by the surgical procedure. Modifications in this unit may result in the onset of dry eye disease (DED), that consists in “a multifactorial disease of the ocular surface characterized by a loss of homeostasis of the tear film, and accompanied by ocular symptoms, in which tear film instability and hyperosmolarity, ocular surface inflammation and damage, and neurosensory abnormalities play etiologic roles” (3) (Figure 1). Ocular discomfort or burning, foreign body sensation, blurred vision, tearing and light hypersensitivity are the most common and impairing symptoms, while the main signs are conjunctival hyperemia, eyelid margin irregularity, and tear film changes associated with positive Schirmer’s test, reduced Tear Break Up Time (TBUT), and/or meibomian gland dysfunction (MGD) and corneal fluorescein staining. Notwithstanding, the possible discrepancy between the reported symptoms and the detectable signs is proven (Table 1). It is also widely known that patients with preoperative DED are at higher risk of having a more severe DED after cataract surgery than those with a normal preoperative tear film (28, 55). Trattler et al. also observed that the incidence of DED in patients who have to undergo cataract extraction is much higher than previously assumed (56). In light of this, a preoperative complete ophthalmological evaluation, focusing especially on the ocular surface and its health, is mandatory to optimize psychological and clinical outcomes of cataract surgery: a correct diagnostic-therapeutic approach is the key to improving the quality of life of patients (16, 17). Data on the incidence of postoperative DED reported in the literature is controversial: it ranges from 9 (9) to 100% (10). Recent studies found that 33% of patients develop DED after surgery (11), while another study reported a 37.4% incidence following phacoemulsification (12). Indeed, signs and symptoms of DED affect a considerable range of patients (13, 18, 23) and generally occurred as early as 7 days post-phacoemulsification and the severity pattern improved over time (14). The first sign seen is loss of corneal sensitivity: data available in literature documents that it is reduced in the postoperative period because of corneal nerve fiber incision, but investigators do not agree on how long this lasts and whether it is transient. Concerning this, Oh et al. showed a corneal sensitivity decrease 1 day postoperatively, which returned to almost preoperative levels within 1 month (18), while Kim et al. observed a restoration of preoperative values 3 months after surgery. On the other hand, Khanal et al. reported an alteration after 3 months, not recovering to pre-operative values (19). Lyne even demonstrated irreversible loss of corneal sensitivity at the corneal incision 2 years after surgery in 90% (20). In light of this, more studies are needed that unravel this knot. In contrast, the decline in goblet cell density is a consensus finding: the count is irreversibly altered and is related to the onset and worsening of DED symptoms (18, 22, 23). Postoperative topical therapy also appears to play a role in modifying GCD: corticosteroids would increase it (24), while NSAIDs would decrease it (25). Similar to GCD, a large proportion of patients undergoing cataract surgery had low values of the Schirmer test and TBUT. Again, there is no agreement on a maximum time for recovery and restoration of normal values: indicatively, within 6 months after surgery (23, 26–28). As these two factors, corneal fluorescein staining appears to worsen in the postoperative period (26, 29). On the duration of this worsening, however, data is conflicting: some studies have demonstrated that it continues for months, while others have observed it increases up to 7 days postoperatively and then decreases over the next 2 months (14, 23). In contrast to the parameters just mentioned, tear osmolarity apparently rises from basal values, compromising ocular surface physiology (30), but few studies have currently been conducted on this. Moreover, it is proven that meibomian gland dysfunction (MGD) plays an important etiopathogenetic role in DED post cataract surgery. Lipid layer thickness appears to be the most crucial factor to manage in the pre- and postoperative phases. Indeed, lipid-based tear substitutes could be the most suitable in these cases: on this topic, Miháltz et al. revealed that lipid-containing artificial tears appear to be superior to those containing sodium hyaluronate in patients with significant MGD (46). In addition to artificial tears, it has been shown that anti-inflammatory management is effective in alleviating postoperative MGD. However, the preoperative management had significantly better meibomian glands outcomes than postoperative enhanced anti-inflammatory treatment: analyzing 20 studies including 2,247 eyes preoperatively and 1 month after surgery, Lu et al. observed that patients with pre-existing MGD had a worsening of subjective symptoms and quantitative indices (e.g., BUT) of dry eye after the operation, whereas in the general population the symptoms remained unchanged and BUT decreased slightly after surgical procedure (57). So, based on these findings, the preoperative evaluation and management could reduce meibomian gland impairment after cataract surgery (33). Confirming this, a randomized clinical trial demonstrated that using a warm compress associated with eyelid massage for 20 minutes preoperatively determined a higher postoperative TBUT than using postoperative drops alone (39). Park et al. also observed how preoperative Lipiflow treatment could prevent and limit MGD and dry eye induced by surgery (31). As far as surgery is concerned properly, some studies also investigated the difference between the two main surgical techniques, femtosecond laser cataract surgery (FLACS) and traditional cataract surgery, with ocular surface symptoms, but there were conflicting results about that. Some studies, as shown by Yu et al. (35) and Ju et al. (36), reported that ocular surface symptoms develop at a higher intensity after FLACS than after cataract surgery, probably due to the traumatic effect induced by femtolaser suction on the ocular surface (36, 37), whereas in the study of Schargus et al., this difference was not statistically significant (38). Moreover, studies have shown that phacoemulsification has a lower incidence of postoperative DED than small incision cataract surgery (SICS). This is due to the larger sclero-corneal incision in SICS, which leads to greater corneal denervation and tear instability. Indeed, corneal nerve damage caused by corneal incision may reduce tear production and affect normal evaporation, which could lead to inflammation of the ocular surface: this results in more severe and longer signs and symptoms of DED (9, 18, 58). Even in our opinion, in relation to our clinical experience, the correct intraoperative approach of cataract surgery is pivotal: in particular, the corneal incision might be posterior limbal and temporal (no longer than 2 millimeters to reduce corneal nerve damage). In high-risk cases, it is important to assess the appropriateness of creating a sclerocorneal or posterior limbal corneal incision, as well as the choice of topical, peribulbar, or intraocular anesthesia. During cataract surgery, useful steps that can be taken perioperatively to limit the occurrence of DED reducing irritation of corneal nerve fibers, such as placing viscoelastic on the cornea rather than continuously irrigating with balanced salt solution (BSS) (39, 40) and/or injecting Mydrane into the anterior chamber (41) Interesting to note how operating microscope light exposure time and cumulative dissipated energy (CDE) may be correlated with ocular surface signs and symptoms after cataract surgery (13), However, these results found by Sahu et al. were not statistically significant, so they could be considered as preliminary data and further studies are certainly needed (13). The instillation of perioperative anesthetic eyedrops (42) and the povidone-iodine application on the eye surface (43) is also toxic for the ocular surface and must be taken into account when choosing the therapeutic strategy to limit DED symptoms in the postoperative period. The mainstay treatment in the management of DED consists of tear substitutes and eyelid hygiene, anti-inflammatory agents, tear stimulants, autologous serum, antibiotics, and other therapies (e.g., physical treatments such as warm compresses, complementary medicines such as herbal products, punctal occlusion, and surgical approaches) (59, 60). The instillation of tear substitutes after cataract surgery is controversial: while no significant effectiveness has been demonstrated for saline and tear lubricants (19), Cagini et al. showed the efficacy of trehalose/sodium hyaluronate eye drops in reducing DED factors and improving tear film stability after cataract surgery (44). In addition, Mencucci et al. also documented a good action of sodium hyaluronate 0.1% and carboxymethyl cellulose 0.5% ophthalmic solution in limiting DED symptoms and improving the clinical outcome after cataract surgery (45). Stefan and Dumitrica observed that postoperative anti-inflammatory treatment could also impact the production and stability of ocular tear film. Additionally, they examined the effects of propylene glycol-hydroxypropyl guar (PG-HPG) nanoemulsion lubricant eye drops and observed increased patient satisfaction when used on a healthy ocular surface (45). Confirming this, Srinivasan and Williams reported how lubricating eye drops, PG-HPG nanoemulsion, increase the stability of the tear film and lipid layer thickness, thus helping to restore ocular surface integrity and alleviating the DED symptoms (49). Park et al.’s research also reported a faster restoration of corneal changes when tear substitutes are instilled post-operatively (31); therefore, the default use of artificial tears as prophylaxis of postoperative DED could be a successful strategy. Moreover, for our ophthalmology surgical clinical experience, eye drops, especially PG-HPG nanoemulsion lubricant one, prevent the signs and symptoms of tear film insufficiency, decrease post-surgery complications, and improve epithelial repair. If tear substitutes and palpebral hygiene represent the first therapeutic line for DED, topical medications are the second therapeutic line, including corticosteroids. They are indicated in the management of moderate to severe DED and are most effective when administered in preservative-free formulation (54) and in combination with tear lubricants (61). Regarding this, data suggests a trigger and/or exacerbating activity of preservatives in postoperative DED. About NSAIDs, evidence in literature is instead controversial: according to some studies, they would reduce GCD (25), retard corneal wound healing (62), and would not be superior to topical corticosteroids (34). Other authors, on the other hand, claim that their administration alone or in combination could be safer and more effective than corticosteroids (63). New studies are needed to settle this controversy. Another effective therapy is cyclosporine A (CsA): Hamada et al. demonstrated its beneficial properties in the management of ocular surface health after cataract surgery (52), as did Chung et al. (64). Moreover, they observed the good function of this component in corneal sensitivity restoration (52, 64). It was confirmed by Kang et al., who described the improvement of TBUT and LLT using 0.05% CsA in the postoperative period compared to 0.5% carboxylmethyl cellulose (CMC) in their study (53). Recently, Diquafosol sodium (DQS) has been available for DED treatment. It is a P2Y2 receptor agonist stimulating aqueous and mucin production as well as epithelium repair. Zhang et al. found that 3% DQS improved ocular surface conditions after cataract surgery in both the short and long term (49). Moreover, Kim et al. found that the postoperative TBUT and lipid layer thickness significantly increased in the DQS group compared with the sodium hyaluronate (HA) group (50, 51). However, most of the drugs described above can establish drug-induced epithelial toxicity, which promotes the onset and/or worsening of postoperative DED. Labetoulle et al. suggest avoiding NSAIDs and using preservative-free eye drops without benzalkonium chloride (BAK) before surgery. They also recommend using antiseptics instead of antibiotics, as antiseptics have less corneal epithelial toxicity and reduce the occurrence of antibiotic resistance (65). These recommendations are known and implemented in daily clinical practice. A table summarizes the pre-, intra-, and postoperative measures considered important for reducing the occurrence of DED after cataract extraction (Table 3). We share these insights and apply them in our daily clinical practice. However, further studies are necessary. Further studies are also needed to examine the correlation between ‘cataract surgery’ and ‘hyper lacrimation,’ as well as the correlation between ‘cataract surgery’ and ‘conjunctival sensitivity’; our research did not yield significant evidence on these topics.

**Table 3:** Pre-, intra-, and post-operative measures and aspects to take into account for limit DED after cataract surgery.

Measures to limit DED onset or worsening after cataract surgery		
Preoperative	Intraoperative	Postoperative
Preoperative comprehensive anamnesis	Limit topical anesthesia	Preservative-free antibiotic-corticosteroid association
Schirmer’s test	Reduce irrigation with BSS	Schirmer’s test
Tear break up time (TBUT)	Use viscoelastic on the cornea	Tear break up time (TBUT)
Corneal staining evaluation	Create small corneal incision	Corneal staining evaluation
Corneal alterations (e.g., dystrophies)	Use of Mydrane	Corneal sensitivity evaluation
Meibomian glands evaluation	Faster operating times	Meibomian glands evaluation
Tear lubricants and eyelid hygiene	Limit exposure to microscope light	Use tear lubricants for 3 months
Instillation of topical corticosteroids if moderate DED is present	Remove iodopovidone as best as possible	
Avoid NAIDs	Prefer preservative-free antibiotics	Add lubricant gel if necessary
Prefer antiseptics to antibiotics		

## 5 Conclusion

Our research involves gathering data from literature on changes in tear film homeostasis in patients undergoing cataract surgery, as well as strategies for managing this condition before and after the procedure. Rehabilitation of postoperative visual acuity is the main goal, but postoperative DED increasingly affects vision quality and patient satisfaction. Far from being exhaustive of the topic, our review summarizes the most common alterations characterizing DED after cataract surgery and the most supported evidence published in the literature in terms of pre- and postoperative management to reduce the incidence and severity of DED. However, data on these topics is often discrepant. It is essential to minimize post-operative DED risk with accurate preoperative diagnosis and therapy, though patient-reported symptoms should always be considered alongside visible signs. Surgical aspects (topical anesthesia, femto-laser assisted surgery, duration of surgical time, size of corneal incision, exposure to microscope light, use of iodopovidone and postoperative antibiotic eye drops) appear to significantly influence the incidence of postoperative DED. However, specific tear substitutes, administered preoperatively and postoperatively, can resolve many of the ocular modifications related to cataract surgery. Topical medications can be effective for treating ocular surface disturbances. However, some of these medications, particularly those containing preservatives, may worsen ocular surface disorders in individuals with pre-existing dry eye disease (DED). In conclusion, multicenter randomized trials are needed, in our opinion, to strengthen the already published results and to establish diagnostic-therapeutic protocols to cope with this condition.
